# Tumor Hybrid Cells: Nature and Biological Significance

**DOI:** 10.3389/fcell.2022.814714

**Published:** 2022-02-15

**Authors:** Maria S. Tretyakova, Ayalur R. Subbalakshmi, Maxim E. Menyailo, Mohit Kumar Jolly, Evgeny V. Denisov

**Affiliations:** ^1^ Laboratory of Cancer Progression Biology, Cancer Research Institute, Tomsk National Research Medical Center, Russian Academy of Sciences, Tomsk, Russia; ^2^ Cancer Systems Biology Laboratory, Centre for BioSystems Science and Engineering, Indian Institute of Science, Bengaluru, India

**Keywords:** cell fusion, tumor hybrid cell, metastasis, drug resistance, cancer

## Abstract

Metastasis is the leading cause of cancer death and can be realized through the phenomenon of tumor cell fusion. The fusion of tumor cells with other tumor or normal cells leads to the appearance of tumor hybrid cells (THCs) exhibiting novel properties such as increased proliferation and migration, drug resistance, decreased apoptosis rate, and avoiding immune surveillance. Experimental studies showed the association of THCs with a high frequency of cancer metastasis; however, the underlying mechanisms remain unclear. Many other questions also remain to be answered: the role of genetic alterations in tumor cell fusion, the molecular landscape of cells after fusion, the lifetime and fate of different THCs, and the specific markers of THCs, and their correlation with various cancers and clinicopathological parameters. In this review, we discuss the factors and potential mechanisms involved in the occurrence of THCs, the types of THCs, and their role in cancer drug resistance and metastasis, as well as potential therapeutic approaches for the prevention, and targeting of tumor cell fusion. In conclusion, we emphasize the current knowledge gaps in the biology of THCs that should be addressed to develop highly effective therapeutics and strategies for metastasis suppression.

## Introduction

Metastasis accounts for most cancer fatalities and is the least understood stage of tumor progression. The process of metastasis consists of a series of linked and sequential steps: invasion, intravasation, survival during circulation, extravasation, the establishment of micrometastases, and the growth of macrometastases (van Zijl et al., 2011). The underlying mechanisms by which cancer cells acquire the ability to escape the primary tumor site, migrate to distant locations, and reestablish tumorigenesis are not completely understood.

According to the “seed and soil” hypothesis, metastasis is governed by interaction and cooperation between the cancer cells (seeds) and the host organ (soil) (Akhtar et al., 2019). However, the occurrence and survival of such seed cells are extremely low. Therefore, other mechanisms outside of the “seed and soil” hypothesis may be involved in metastasis.

Cell fusion is a process in which two or more cells fuse and become one due to a common membrane. Cell fusion leads to tumor hybrid cells (THCs) with a common genotype of parental cells (LaBerge et al., 2017) but with novel molecular features (Zhang et al., 2019a). Hybrid cells derived from cancer cells or cancer and normal cells show increased proliferation, drug resistance, decreased apoptosis rate, and avoiding immune surveillance (Li et al., 2014; Gast et al., 2018; Lartigue et al., 2020). THCs also possess increased migration and invasion and an extremely high metastatic potential (Zhang et al., 2019a). Abnormal division of tumor fusion cells may result in the appearance of polyploid cells (Raslova et al., 2007), contributing to genetic complementation by restoring the loss of gene function and the promotion of the survival of such hybrid cells (Duncan et al., 2009; Miroshnychenko et al., 2021). However, to date, there is scarce information about genetic changes driving tumor cell fusion, the life circle of THCs, and the molecular properties that THCs acquire.

In this review, we discuss the factors and mechanisms involved in the occurrence of THCs, their types and role in cancer drug resistance and metastasis, and potential therapeutic approaches for the prevention and targeting of tumor cell fusion. In conclusion, we emphasize the current knowledge gaps in the biology of THCs that should be addressed to develop highly effective therapeutics and strategies for metastasis suppression.

## Tumor Hybrid Cells Formation: Factors and Mechanisms

The mechanisms governing tumor cell fusion are poorly understood. Under normal conditions, fusion events are rare but increase dramatically in pathological conditions such as tissue injury and inflammation (Davies et al., 2009). The pro-inflammatory cytokine tumor necrosis factor-α (TNF-α) is a potent trigger of cell fusion (Mohr et al., 2015). TNF-α induces fusion between M13SV1-Cre breast epithelial cells and MDA-MB-435-pFDR1 triple-negative breast tumor cells through the involvement of the matrix metalloproteinase MMP9 (Weiler et al., 2018). MMP9 also participates in the fusion of macrophages (MacLauchlan et al., 2009), but the exact mechanisms are unclear. Potentially, MMP9 may be associated with the destruction of extracellular matrix components due to proteolytic activity, thereby facilitating the interaction of cell membranes (MacLauchlan et al., 2009). Poor vascularization, resulting in hypoxia and deficient access to nutrients, can be another trigger for tumor cell fusion. For example, the fusion between mesenchymal stromal cells (MSCs) and breast tumor cells is significantly increased in hypoxic conditions (Noubissi et al., 2015).

Cell fusion is promoted by fusogenic proteins. These proteins assemble into unilateral or bilateral complexes, which determine the site of cytoplasmic membrane fusion and overcome the energy barriers. The main fusogens are syncytins, which play a key role in developing human placental syncytiotrophoblasts (Zhu et al., 2020). Syncytin1 (Syn1) belongs to the human endogenous retrovirus (HERVs) family. This protein participates in human placental morphogenesis (Mi et al., 2000) and plays an essential role in the fertilization of gametes (Bjerregaard et al., 2014). Syn1 and annexin A5 are upregulated in prostate cancer (PC3) and muscle (hMYO) cell cocultures and are involved in tumor cell fusion (Uygur et al., 2019). Syn1 is also upregulated in MCF-7 luminal and MDA-MB-231 triple-negative breast cancer cell lines and about 38% of breast tumor specimens and facilitates the tumor cell fusion with endothelial cells while blocking its expression inhibits the formation of THCs (Bjerregaard et al., 2006).

Viruses are another trigger for cell fusion (Kanai et al., 2019; Leroy et al., 2020), facilitating merging infected and uninfected cells through the production of viral fusogens (Chan et al., 2020; Vance and Lee, 2020). For example, the measles virus causes fusion between normal and lung cancer cells, but hybrids show signs of cellular senescence (Chuprin et al., 2013).

When fusogens are not activated, or the fusion mechanism remains unknown, and the actin skeleton can serve as another driver of the occurrence of THCs. Examples include the formation of actin protrusions that promote the fusion of macrophages and myoblasts (Faust et al., 2019). For instance, myoblasts utilize actin-propelled membrane protrusions to promote fusogenic protein engagement and fusion pore formation. Invasive protrusions trigger a MyoII-mediated mechanosensory response in a cell fusion partner (Kim et al., 2015).

The phosphatidylserine (PS), a membrane phospholipid, is also involved in cell fusion (Birge et al., 2016; Kang et al., 2020). A link between cell fusion and PS exposure was first described in the formation of skeletal muscle fibers (van den Eijnde et al., 2001). It is assumed that membrane remodeling in cell fusion depends on PS and phosphatidylserine-recognizing proteins (Whitlock and Chernomordik, 2021). The link between PS and cell fusion is not limited to normal cells. PS is often overexpressed on the surface of cancer cells including different cell lines (MDA-MB-231-Luc-D3H2LN, Gli36, and U373), which may indirectly indicate the ability of these cells to fusion (Vallabhapurapu et al., 2015; Sharma and Kanwar, 2018).

Cell fusion depends on the type of cancer and normal cells, as observed in the co-culture of human glioblastoma U87 and U373 cells with MSCs. In particular, more hybrids were formed from U87 cells than from U373 cells. The underlying mechanisms are not yet clear and most likely related to specific gene expression features of these types of glioblastoma cells (Oliveira et al., 2018). Further research, e.g., using different omics approaches, may reveal genetic and molecular factors that govern glioblastoma cell fusion with MSCs. The cell-dependent capacity to form THCs is also seen in breast cancer cell lines. For example, MCF-7 luminal breast cancer cells are more prone to forming hybrids than triple-negative breast cancer cells—MDA-MB-231 and SUM159 (Miroshnychenko et al., 2021).

**FIGURE 1:**
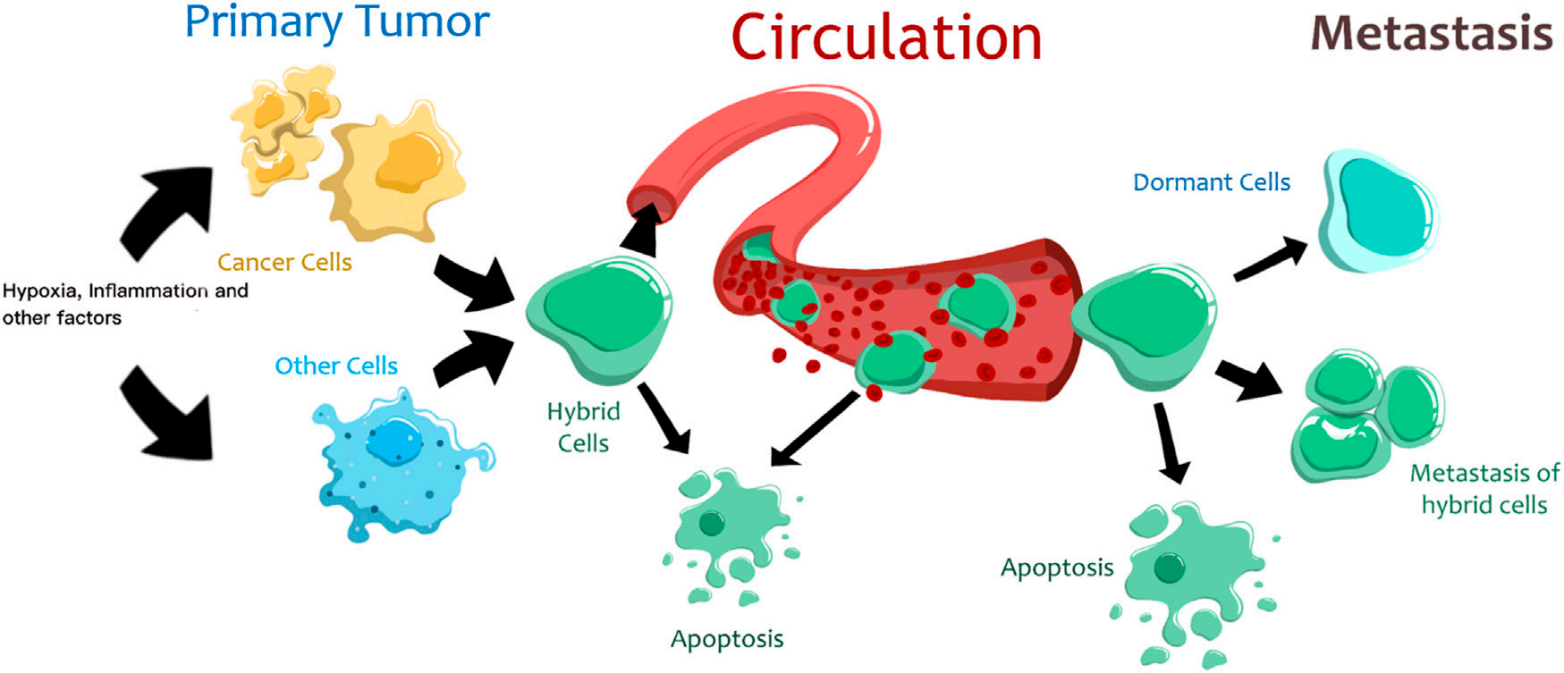
The proposed life cycle of tumor hybrid cells. Different factors trigger the fusion of cancer and normal cells. Most hybrid cells die, and only a few can reach distant organs. These hybrid cells can enter a state of dormancy or form metastases.

Thus, tumor cell fusion is a complex regulated process initiated by different external and internal factors (Figure 1). However, it should be noted that the resulting hybrid cells have low viability, and only a few of them survive and acquire new properties. This is due to post-fusion processes that are accompanied by improper segregation of chromosomes, impaired proliferation, or cell death (Sieler et al., 2021). After fusion, some THCs remain multinucleated, others undergo a transition “from heterokaryon to syncarion” or a decrease in ploidy (Hass et al., 2021). Although most THCs usually become more aggressive than the parental cells, cancer cell aggressiveness may also decrease after fusion (Staroselsky et al., 1991). Eventually, only some THCs survive in the primary tumor, penetrate the circulation, reach distant sites, and enter dormancy or form metastatic lesions (LaBerge et al., 2021; Melzer et al., 2021).

However, what changes occur in the cells before fusion, what signaling pathways are induced by cell fusion drivers, and what processes are activated and suppressed in THCs are currently unknown. These and other questions need to be answered, and a lot of work remains to be done in the future to understand more deeply the mechanisms of THC formation.

## Types of Tumor Hybrid Cells

**FIGURE 2:**
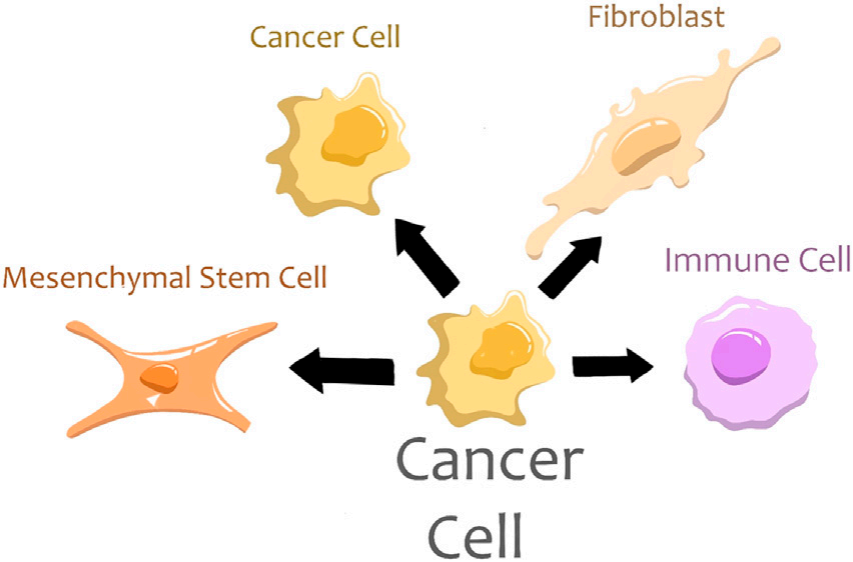
Types of tumor hybrid cells.

Cancer cells may fuse with other cancer cells and different normal cells such as immune cells, MSCs, and fibroblasts (Figure 2). All these fusion types result in an acquisition of new features and an increase in the aggressiveness of tumor cells.

### Fusion Between Cancer Cells

Cancer cells tend to fuse with other cancer cells regardless of the type of cancer. Such fusion can afford cancer cells to change genomic material faster and much larger scale than genetic mutations. The resulting hybrid cells acquire new properties such as drug resistance and increased metastatic potential (Miller et al., 1989; Lu and Kang, 2009; Yan et al., 2016; Searles et al., 2018; Miroshnychenko et al., 2021).

### Fusion Between Cancer and Immune Cells

The fusion of leukocytes and cancer cells can initiate metastasis. This theory was proposed more than a century ago by Prof. Otto Aichel (Warner, 1975). Recently, various studies have provided evidence showing that the fusion of cancer cells and leukocytes leads to metastasis. Leukocyte-cancer cell fusions are frequently observed in lymph node metastases of patients who underwent bone marrow transplantation operations. These hybrid cells contain a mixture of donor and recipient DNA (Chakraborty et al., 2004; Yilmaz et al., 2005; LaBerge et al., 2017).

Macrophages are divided into two main types: M1 and M2. M1 macrophages are pro-inflammatory and characterized by the release of inflammatory cytokines. M2-macrophages are considered anti-inflammatory and associated with releasing interleukins (IL)-4, IL-13, and IL-10, and promote tumor growth and progression (Yin et al., 2016; Zhu et al., 2017; Nie et al., 2019). Some studies showed that cancer cells fuse with M2 macrophages (Shabo et al., 2015; Kuwada et al., 2018). However, it is not clear at what point the macrophage acquires M2 polarization before or after fusion with cancer cells. Fusion between cancer cells and M2 macrophages may lead to the formation of hybrid cells with stem CD44^+^ CD24^-/low^ phenotype and features of the epithelial-mesenchymal transition. These THCs are characterized by aggressive demeanor, including increased migration, invasion, and tumorigenicity, but have reduced proliferation compared to parental cells (Ding et al., 2012). The appearance of such stem-like THCs may enhance heterogeneity of cancer stem cells in particular and intratumor diversity in general.

However, the place of fusion of cancer cells and macrophages remains an open question. Does it occur in the blood, the tumor, or the lymphatic system? The impact of cancer-macrophage fusion cells on the immune system is also unclear and is of great interest for future research.

### Fusion between Cancer Cells and MSCs

Cancer cells can merge with MSCs and thus increase their malignant potential (Yong Wang et al., 2012). Several studies *in vitro* and *in vivo* showed that MSCs fuse with breast cancer cells and novel hybrids possess more aggressive properties than parental cells (Melzer et al., 2018a; Melzer et al., 2018b; Melzer et al., 2019). Such THCs contribute to cell plasticity and heterogeneity in tumorigenic potential and chemotherapeutic responsiveness (Melzer et al., 2021). MSCs can fuse with cancer cells spontaneously (Xue et al., 2015) and under the influence of external factors, such as hypoxia. The spontaneous fusion of MSCs and lung cancer cells leads to the formation of slow-growing hybrids with non-carcinogenic features but with EMT and stem-like phenotype (Wei et al., 2014; Xu et al., 2014). Hypoxia-related breast cancer-MSC fusion promotes the formation of hybrid cells with increased migration ability and stem features that may enable their dissemination to distant sites (Noubissi et al., 2015).

### Fusion between Cancer Cells and Fibroblasts

Fibroblasts represent the largest population of cells in the tumor environment (Truffi et al., 2020). Cancer-associated fibroblasts are a heterogeneous and highly plastic group of cells that are one of the key players in the regulation of metastatic cascade (Suetsugu and Hoffman, 2021). Fusion between fibroblasts and cancer cells is considered part of the tumor-stroma interaction (Vucicevic Boras et al., 2018). Cancer-associated fibroblasts fuse with prostate cancer cells and form hybrid cells in the coculture model. Most tumor fusion cells die, but the surviving ones acquire increased proliferative activity and aggressiveness (Ruoxiang Wang et al., 2012). Moreover, the survived hybrid cells acquire genomic alterations and novel molecular characteristics, which can potentially cause of tumor invasion and metastasis (Lartigue et al., 2020).

Thus, fusion with different cells can be a mechanism of cancer evolution by gaining novel properties to survive under unfavorable hypoxic conditions and pressure from the immune system.

## Role of Tumor Hybrid Cells in Metastasis

Various manifestations of cancer cells forming hybrid cells together with stromal cells have been reported (Table 1). For instance, human mesenchymal stroma/stem-like cells (MSCs) can spontaneously fuse with MDA-MB-231 breast cancer during co-culture to form two different aneuploid populations with varying short tandem repeat (STR) profiles (MDA-hyb1, MDA-hyb2). Both these populations had a higher proliferation rate than MDA-MB-231 cells and enrichment of mesenchymal markers such as FN1, SNAI2, and MMP9. *In vivo* experiments revealed higher tumorigenicity and metastatic potential of both the hybrid cell populations (Melzer et al., 2018b). Another study reported the fusion of human mesenchymal stromal cells with MCF-7 and T74D breast tumor cells (Chitwood et al., 2018), both of which are more epithelial cells compared to MDA-MB-231 (George et al., 2017). Spontaneous cell fusion can also happen *in vivo*, and lung metastases were shown to possess a higher frequency of hybrid cells formed between MSCs and murine fat pad tumor cells (PyVT) when compared to the primary breast tumor, suggesting that hybrid cells can be formed in the primary tumor, metastasize, and then proliferate at the metastatic site (Chitwood et al., 2018). Similar traits are reported on the fusion of mesenchymal cells with those of the same (mesenchymal) lineage. The fusion of fibroblasts IMR90 with precancerous cells of the same lineage (IMR90 E6/E7) led to genetically unstable clones, each of which was more aggressive, and metastatic than their parental populations. Reinforcing observations were made upon sarcoma cell fusion hybrids. When engrafted in mice, hybrid cells formed tumors reminiscent of undifferentiated pleomorphic sarcoma (UPS) in terms of genetics, clinical behavior, and tumor histology (Lartigue et al., 2020). Together, these observations highlight the increased metastatic potential of tumor hybrid cells.

**TABLE 1:** Summary of reports showing the fusion of different cancer cells with immune and stromal cells and their impact on EMT, CSCs, and metastasis.

Cancer cell	Stromal cell	Fusion	Migration/invasion assay	CSC characterization (surface markers/Functional assay done)		References
Human H460 and A549 non-small-cell lung carcinoma lines	Monocytes	<4%	Transwell migration	Nanog, Oct3/4, KLF4, Sox2, MYC	Formation of spheroid aggregates	Aguirre et al. (2020)
T47D human breast cancer cells	Human mesenchymal stromal cells		Timelapse microscopy			Chitwood et al. (2018)
Peripheral blood from pancreatic ductal adenocarcinoma patients, MC38 mouse intestinal epithelial cancer cells	Macrophages	0.48%	Boyden chamber invasion assay			Gast et al. (2018)
IB105/106 sarcoma cell lines	IMR90 fibroblasts		Scratch assay, Boyden chamber invasion assay		Soft agar colony formation assay	Lartigue et al. (2020)
Human MDA-MB-231 breast cancer cells	Mesenchymal stroma/stem cells			CD29, CD44, CD73, CD90, CD105, CD146, CD166	*In vivo* tumorigenicity	Melzer et al. (2018a)
Human MDA-MB-231 breast cancer cells	Mesenchymal stroma/stem cells	0.015–0.18%		CD44, CD73, CD90, CD105	*In vivo* tumorigenicity	Melzer et al. (2019)
IMR90- E6E7 HRAS^G12V^	IMR90- E6E7 fibroblasts	2%	Scratch assay	ALDH, NANOG and OCT4	Sphere formation assay	Merle et al. (2021)

Higher heterogeneity has been proposed to underlie this metastatic propensity of hybrid cells (Gast et al., 2018). Hybrid cells formed *in vitro* by the fusion of bone marrow-derived macrophages with murine cells MC38 (intestinal epithelial cancer) and B16F10 (melanoma) possessed fused nuclei exhibiting neoplastic transcriptional identity, while notably, retained macrophage gene expression signatures. They also exhibited contact inhibition and mesenchymal histologic sheet-like features. *In vivo*, hybrid cells are a rare subpopulation (0.03–0.69%), but have a shorter doubling time and enhanced metastatic ability. In patients, circulating hybrid cells (CHCs) were identified based on co-expression of CD45 (leukocyte marker) with CK (cytokeratin: epithelial marker). The frequency of CHCs, but not that of CTCs (circulating tumor cells: CD45-negative, CK-positive), was observed to be a prognostic marker for patient survival, regardless of disease stage. Intriguingly, fused cells demonstrated a combination of adhesion biases, as identified by a microenvironment microarray (MEMA) platform (Gast et al., 2018). Such broader adhesive affinity may offer a survival advantage during metastatic cascade, although direct experimental validation of this hypothesis remains to be yet established.

Another aspect of metastatic fitness observed in tumor hybrid cells is the acquisition of EMT and/or stemness, two interrelated traits that can accelerate metastasis (Celià-Terrassa and Jolly, 2020). MCF-7 cells, when fused with macrophages, had upregulated Snail1, Snail2, and Vimentin and decreased E-cadherin levels (Ding et al., 2012). Upon fusion with macrophages, both MCF-7 and MDA-MB-231 cells had a higher percentage of CD44^+^/CD24^−^cells and enhanced tumorigenicity *in vivo*, suggesting that cell fusion can also be a source of cancer stem cells (CSCs). Similar observations are reported in the fusion of lung cancer cells (Xu et al., 2014; Zhang et al., 2019b) and gastric epithelial cells (He et al., 2015). Interestingly, the traits of MCF-7/macrophage hybrid cells, such as expression of M2 macrophage marker CD163, could not be explained by paracrine engagement of MCF-7 cells with macrophages (Shabo et al., 2015). Further, breast cancer patients with >25% of cancer cells expressing CD163 had worse disease free and recurrence free survival than those with <25% of such cells. Besides displaying EMT and stemness, THCs can also be immune-evasive, and their frequency in circulation can indicate the metastatic ability of a tumor (Aguirre et al., 2020).

The abovementioned studies offer putative mechanistic insights into earlier phenomenological observations on increased aggressive behavior of somatic cell fusion events, as reported in many cancers (Sidebottom and Clark, 1983; De Baetselier et al., 1984; Pawelek, 2000; Pawelek and Chakraborty, 2008; Sieler et al., 2021). Cell-cell fusion can lead to DNA exchange (Searles et al., 2018), which can drive aneuploidy and increased genetic and phenotypic heterogeneity, thus aggravating cancer progression.

## Role of Tumor Hybrid Cells in Drug Resistance

Besides mediating metastasis, THCs can also drive resistance against various drugs. In a metastatic colon carcinoma model *in vivo* (Carloni et al., 2013), fused cells were found to be resistant to both 5-fluorouracil (5-FU) and oxaliplatin. Similarly, hybrid cells formed due to fusion of two sister subpopulations—168FAR and 44FTO—was found to be more resistant to both melphalan and methotrexate than either parental subpopulation. Also, these two co-existing subpopulations had varied organotrophic behavior; the hybrid clone showed spontaneous metastatic traits (Miller et al., 1989). Beyond chemoresistance, the spontaneous fusion between M2-macrophages and MCF-7 cancer cells can drive radioresistance *in vitro*; such hybrid cells have enhanced DNA repair capacity and less heterogeneity in DNA damage upon exposure to radiation (Lindström et al., 2017). While *in vivo* existence of these radioresistance traits remain to be observed, intriguingly, and radiation itself can drive homotypic cell fusion of a subpopulation of glioblastoma cells (Kaur et al., 2015). These pre-existing radiation-resistant mononucleated cells can survive radiotherapy by arresting the cell cycle and repairing their damaged DNA. They undergo cell-cell fusion to form multinucleated giant cells (MNGCs).

EMT and/or stemness have been often associated with resistance to many chemotherapeutic drugs, radiation therapy, targeted therapy, and immunotherapy across multiple cancers (Peijing Zhang et al., 2014; Zheng et al., 2015; Dongre et al., 2017; Mal et al., 2020; Sahoo et al., 2021a; Sahoo et al., 2021b ), but whether these processes are responsible for aggressive behavior of THCs remains to be elucidated. Further analysis of any causal contribution of these processes can be performed through dissecting the heterogeneity of THCs seen in primary tumor and/or circulation. For instance, a comparison of hybrid clone cells formed by spontaneous fusion events of human M13SV1-EGFP-Neo breast epithelial cells and HS578T-Hyg, MDA-MB-435-Hyg, and MDA-MB-231-Hyg cancer cells demonstrated that fusion cells formed by HS578 cells had stronger *in vitro* tumor-initiating traits as compared to those formed by MDA-MB-231 and MDA-MB-453 cells, as identified by surface marker (frequency of ALDH1+ cells) and functional (mammosphere) assays (Fahlbusch et al., 2020). Similarly, hybrid cells in circulation were shown to reflect the heterogeneity of both epithelial and non-epithelial malignancies and can be used as a translational non-invasive readout of tumor aggressiveness, given their higher frequency than that of CTCs (Dietz et al., 2021). Hybrid clones can also be metabolically heterogeneous, as noted for subpopulations of CSCs with varying EMT phenotypes (Luo et al., 2018), but overall, they display an enriched Warburg-like phenotype (upregulated glycolysis) (Brito et al., 2021). Cell-to-cell heterogeneity has been shown to accelerate tumor progression and/or metastasis in various contexts, such as cooperation between EMT and non-EMT cells (Tsuji et al., 2008; Neelakantan et al., 2017), but whether such cooperation is seen among tumor hybrid cells and whether any such non-cell-autonomous behavior has a functional role to play in enhancing metastasis remains to be identified *in vitro* and *in vivo*.

It should be noted that not every instance of cell fusion necessarily implies a higher metastatic and/or chemoresistant set of features. For example, MDA-hyb3 cells formed by *in vivo* spontaneous fusion of MSCs with MDA-MB-231 cells had reduced tumor-forming and metastatic ability compared to MDA-MB-231 cells, despite having enhanced proliferation (Melzer et al., 2019). Similar observations were noted for SK-MSC-hyb1 and -hyb2 ovarian cancer hybrids (fusion of SK-OV-3 human ovarian cells with MSCs) (Melzer et al., 2020). A detailed molecular mapping of such less metastatic hybrid cells remains to be done. Therefore, a comparative analysis of more vs. less metastatic and aggressive hybrid clones will be essential to identify promising therapeutic vulnerabilities that may be clinically relevant.

## Therapeutic Options for Targeting Tumor Hybrid Cells

**FIGURE 3:**
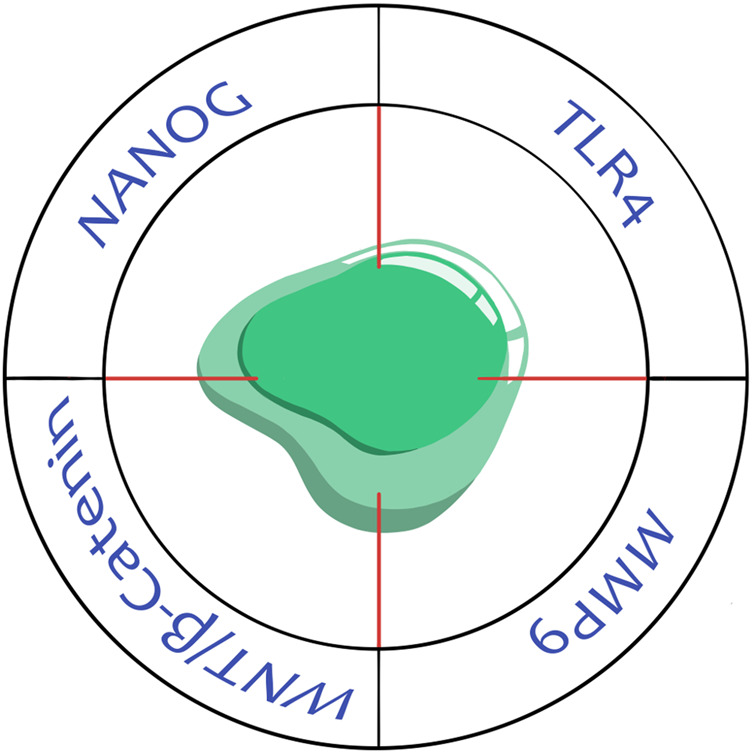
Potential therapeutic targets in tumor hybrid cells. TLR4, toll-like receptor 4; MMP9, matrix metalloproteinase 9; Wnt/β-catenin, signaling pathway; Nanog, transcription factor.

The prevention of tumor cell fusion and targeting THCs seem attractive for anti-cancer treatment, particularly a decrease in therapy resistance and the suppression of metastasis. Two main approaches can be distinguished to affect neoplastic and nonneoplastic components using different molecules as potential therapeutic targets (Figure 3).

Matrix metalloproteinase MMP9 is a critical molecule for the TNF-α-induced fusion of breast epithelial (M13SV1) and triple-negative breast tumor cells (MDA-MB-435) (Weiler et al., 2018). The suppression of MMP9 by SB-3CT (MMP2/9 chemical inhibitor) leads to a decreased fusion rate of these cells (Saito et al., 2015). Likewise, tetracycline-based antibiotic minocycline is effective in targeting MMP9 and blocking the TNF-α-induced fusion frequency of M13SV1 and MDA-MB-435 cells (Weiler et al., 2018).

Toll-like receptors (TLRs) belong to the pathogen recognition receptors that play a crucial role in the innate immune system. TLR4-mediated signaling is implicated in tumor cell invasion, survival, and metastasis in various cancers (Yang et al., 2014). TLR4 and TLR9 are highly expressed in M13MDA-435-1 and -3 hybrid breast tumor cells (Fried et al., 2016), while their cultivation in the presence of the TLR4 ligand lipopolysaccharide induces apoptosis in all hybrid clones (Fried et al., 2016).

The activation of the Wnt/β-catenin signaling pathway is crucial for the artificial fusion between breast tumor cells (N2O2) and macrophages using polyethylene glycol hydrogels and the promotion of proliferation, migration, invasion, and colony formation of THCs (Zhang et al., 2019a). In turn, XAV-939, a small molecule inhibitor of the Wnt/β-catenin signaling pathway, can reduce the capability of fusion of macrophages with tumor cells leading to a decrease in cell proliferation, migration, and invasion (Zhang et al., 2019a).

Cell fusion modifies epigenetic landscape unlocking the expression of transcription factors, for example, Nanog, which is directly involved in cell reprogramming. It was shown that Nanog is strongly expressed by spontaneous mesenchymal hybrid cells generated from sarcoma cells (B105-DsRed, IB106-GFP, and IB105/106) and fibroblasts (IMR90) (Lartigue et al., 2020; Merle et al., 2021). Inhibition of Nanog by siRNA significantly decreases the migration capacity of THCs (Merle et al., 2021).

Tumor cell-macrophage hybrids express both macrophage (CD14, CD68, CD163, CD204, and CD206) and tumor-specific markers (ALCAM, MLANA, KRT, EpCAM, CXCR4, and CD44) (Clawson et al., 2015). These fusion cells also express integrin subunits (α3, α5, α6, αv, β1, and β3) and GnT-V (β1,6-acetylglucosaminyltransferase-V) (Pawelek, 2005). Therefore, the aforementioned molecules can be potential targets for destroying tumor-macrophage fusion cells (Soltantoyeh et al., 2021). Targeting GnT-V, as well as SPARC, SNAIL, and MITF, in combination with chimeric antigen receptor (CAR) T cells that are specific to surface proteins (β1,6-branched oligosaccharides, MET, and LAMP1) of tumor-macrophage fusion cells, may improve chimeric antigen receptor CAR T cell performance in metastatic melanoma (Soltantoyeh et al., 2021).

THCs themselves can act as antitumor therapeutic options. Purified dendritic cell-tumor fusion hybrids supplemented with the non-adherent dendritic cell population elicit the robust antitumor immune response in breast cancer model *in vitro* (Yunfie Zhang et al., 2014). Similarly, the fusion between dendritic cells and cancer cells generates hybrids that activate both CD4^+^ and CD8^+^ T cells, activating the antitumor immunity (Koido et al., 2014a; Koido et al., 2014b). These results indicate that fusion cell vaccines can effectively induce antigen-specific responses and activate anti-tumor immunity; however, further research is needed to evaluate this phenomenon *in vivo* models*.*

Thus, different therapeutic options can be addressed to prevent tumor cell fusion and destroy THCs. However, the efficiency of all of them was demonstrated *in vitro*, and further studies on *in vivo* and clinical models are required, especially in terms of understanding the mechanisms of THC formation in the multilevel organism systems and the development of instruments for their control.

## Challenges and Current Trends

Cell fusion promotes tumor growth and progression by formating new cells with increased drug resistance, immunotolerance, and metastatic properties. Therefore, the identification of THCs and revealing their molecular features may be effective in developing therapeutic approaches against cancer metastasis. Despite the abundance of studies devoted to THCs, several critical points are unclear.

The problem of identifying THCs remains unresolved. At present, THCs are identified focusing on the specific markers of parental cells. However, fusion cells that lack those markers cannot be categorized as THCs. Therefore, there is an urgent need to identify surface molecules and/or genetic alterations that can act as THC markers, regardless of the parental cells. This challenge is difficult because of the same or largely overlapping genetic content of parental cells and THCs and can be overcome through a deep understanding of the molecular landscape of different types of THCs. For example, comparative analysis of THCs and parental cells using single-cell DNA sequencing combined with oligo-conjugated antibodies or cell sorting followed by DNA sequencing may identify genetic mutations associated with cancer cell fusion. Single-cell RNA sequencing of different tumors followed by bioinformatic analysis of differentially-expressed genes, DNA ploidy, and genetic alterations may identify new types of THCs and their unique features not present in parental cells.

Another unresolved problem is related to the absence of information on the specificity of THCs to various cancers. Due to specific etiology and pathogenesis, different cancers may harbor individual repertoire of THCs. It is also unknown whether the occurrence of THCs depends on the stage and histological and molecular properties of cancers. These questions can be addressed by a comparative analysis of the proportion and composition of THCs between different cancers and their association with histological/molecular type and clinicopathological parameters of a particular type of cancer.

The current data indicate the strong heterogeneity of THCs, which results in cell populations with varying degrees of drug resistance, immune tolerance, and invasive, and metastatic potentials. Together with a diversity of tumor cells, this evidence significantly increases the level of intra- and inter-tumor heterogeneity and thus the chances of treatment failure and poor outcome. Therefore, deciphering cell heterogeneity and identifying the most aggressive cell populations are one of the key aims to uncover the nature of THCs. The questions about genetic alterations that can trigger tumor cell fusion, the molecular landscape of cells after fusion, and the lifetime and fate of different THCs also remain unanswered. In this case, the potential instrument can be the use of single-cell omics and fluorescent protein-based cell tracking technologies, as already mentioned above.

Thus, further research should focus on a comprehensive and multi-omic analysis of THCs to reveal genetic alterations leading to tumor cell fusion and specific markers of THCs, to investigate how strong the molecular landscape of hybrid cells changes compared to parental cells, and to understand the specificity of THCs to various cancers, and their correlation with clinical and pathological parameters.
